# Low-dose colchicine prevents sympathetic denervation after myocardial ischemia-reperfusion: a new potential protective mechanism

**DOI:** 10.2144/fsoa-2020-0151

**Published:** 2020-11-23

**Authors:** Fabien Huet, Jérémy Fauconnier, Marion Legall, Pierre Sicard, Catherine Lozza, Alain Lacampagne, François Roubille

**Affiliations:** 1 Department of Cardiology, Montpellier University Hospital, Montpellier, Occitanie, France; 2 University of Montpellier, CNRS, INSERM, CHRU Montpellier, Montpellier, France

**Keywords:** colchicine, heart rate variability, myocardial infarction, sympathetic innervation

## Abstract

**Purpose:**

To evaluate the impact of colchicine on sympathetic denervation after acute myocardial infarction (AMI).

**Materials & methods:**

Ischemia/Reperfusion was induced in C57BL/6J male mice. Left coronary artery was ligated during 45 min followed by reperfusion. 400 μg/kg of colchicine or the placebo was administrated intraperitoneally 15 min before the reperfusion.

**Results:**

Colchicine treatment significantly improved heart rate variability index after AMI. Colchicine prevented sympathetic denervation in the remote area (p = 0.04) but not in the scar area (p = 0.70).

**Conclusion:**

These results suggest promising protective pathway of colchicine after AMI.

## Lay abstract

This is a preclinical study of acute myocardial infarction in mice treated with colchicine or saline injection. ECG monitoring, immunofluorescence histology and NGF serum level measurement were performed. Here, it is demonstrated that colchicine improves heart rate variability, reduces cardiac denervation. The randomized COLD-MI trial will soon start and include patients. Cardiac denervation will be assessed using nuclear imaging with méta-iodobenzylguanidine (MIBG).

In several preclinical models, acute myocardial infarction (AMI) has been shown to induce local sympathetic denervation [1] leading to an heterogeneity in norepinephrine (NE) release within the heart, predisposing to an electrical instability and arrhythmia in patients after AMI or in chronic heart failure [2]. AMI is accompanied by an inflammatory response which has been demonstrated to worsen reperfusion injuries and participate in the postischemic remodeling. Although anti-inflammatory drugs have been proven to limit reperfusion injuries in preclinical studies, translation to the clinic remains an important challenge [3,4]. Colchicine was shown to reduce neutrophil migration in the ischemic scar, reduce myocardial necrosis and systemic inflammation, limit ventricular remodeling in a mice model of ischemia/reperfusion (I/R) [5], and recently to reduce cardiac events after AMI in patients [6]. Because inflammation after AMI is responsible for sympathetic nervous system remodeling [7], we hypothesized that colchicine could also prevent sympathetic denervation after AMI. Thus, the objective of this study was to evaluate the impact of colchicine on heart rate variability (HRV) and cardiac sympathetic neuron remodeling in a mouse model of AMI.

## Materials & methods

### Animal model

About 8-week old C57BL/6J male mice (25g) were kept on a 12:12h light-dark cycle with *ad libitum* food and water access.

### Surgery

Myocardial I/R injuries were performed under general anesthesia induced with 4% isoflurane and maintained with 2% isoflurane and after orotracheal intubation with a 22G venous catheter for controlled ventilation (Minivent, Harvard Apparatus) with controlled stroke (10 μl/g) and frequency (150/min). After left thoracotomy and muscular dissection, ligation of the left coronary artery was performed with a 8–0 silk and a smooth catheter was applied on the artery to obtain an ischemia for 45 min. About 400 μg/kg of colchicine or placebo was administrated intraperitoneally 25 min before the reperfusion (blinded administration). Muscle and cutaneous plans were sutured with silk 6–0. Sham-operated animals (placebo or colchicine) were subjected to the same surgical procedure without coronary ligation. At 7 days after I/R procedure et after blood puncture, mice euthanasia was performed under general anesthesia, by heart removal.

### *In vivo* telemetry

ECGs were obtained from conscious adult mice using ETA-F20 (Data Sciences International) telemetry implants. ECG parameters (QRS duration, QT duration, heart rate [HR]) were measured using 6 h nocturnal ECGs based on exclusively sinusal complex. For HRV analysis, we measured standard deviation of NN intervals (SDNN), triangular interpolation of RR intervals (TINN), root mean square of the successive differences (RMSSD).

### Immunohistochemistry

Permeabilized myocardial transverse 10 μm sections were incubated with rabbit anti-TH (1:200, Novus Bioscience) and sheep antifibrinogen (1:1000, AbD Serotec) antibodies overnight at 4°C and incubated with the Alexa Fluor 647-conjugated rabbit IgG-specific antibody (1:750; Invitrogen) and Alexa Fluor 555-conjugated sheep IgG-specific antibody (1:750). From each heart, images were acquired from three different sections obtained from the base-to-apex axis. Staining was quantified using threshold discrimination analysis – ImageJ software (https://imagej.nih.gov/ij/).

### Serum sample analysis

Seven days after reperfusion and before animal euthanasia, a retro-orbital blood puncture was performed under pentobarbital anesthesia. The NGF biomarker was measured in serum using a mice ELISA kit (Sigma Aldrich, Saint-Quentin Fallavier, France). The data were revealed by Luminex Multiplex assay (Milliplex^®^ MAP Millipore, MA, USA).

### Statistical analysis

Statistical analysis was performed with Prism (GraphPad software, CA, USA). Groups were compared with a one-way analysis of variance (ANOVA) test. In case of statistical difference, we compared the two groups of mice treated with colchicine or placebo for each analysis uning Mann–Whitney test. The significance was fixed at p < 0.05 (*). All data are expressed as percentage, mean and standard deviation in tables and in standard error of mean in Graphs.

## Results & discussion

### Colchicine improves *in vivo* post ischemic HR variability

**Figure 1.**
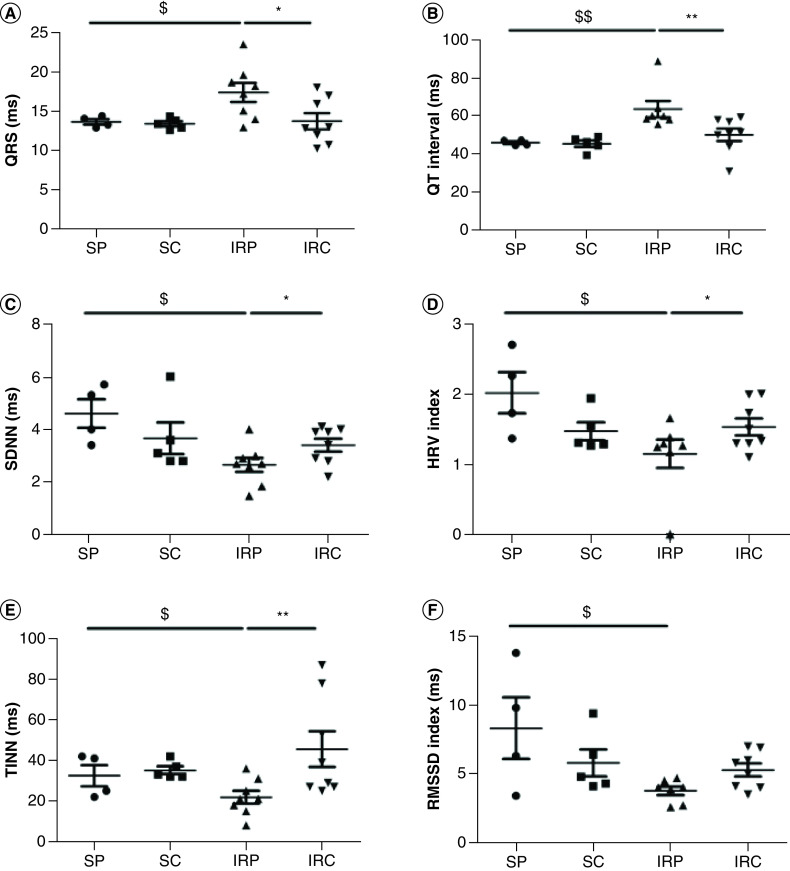
*In vivo* telemetry analysis. QRS duration **(A)** and QT interval **(B)** HRV analysis of SDNN **(C)** HRV index **(D)** TINN **(E)** and the RMSSD index **(F)** HRV; SDNN; TINN; RMSSD; VLF; LF; HF. ^$^Significantly significant in comparison with the sham placebo group ($ for p < 0.05; $$ for p < 0.01). *Significantly significant in comparison with the IR-placebo group (* for p < 0.05; ** for p < 0.01). HF: High frequency; HRV: Heart rate variation; IRC: IR colchicine; IRP: IR placebo; LF: Low frequency; RMSSD: Root mean square of the successive differences; SC: Sham colchicine; SDNN: Standard deviation of successive differences; SP: Sham placebo; TINN: Triangular interpolation of RR intervals; VLF: Very low frequency.

*In vivo* telemetry acquisition was performed in vigil mice for 24 h, 6 days after AMI, where other studies have shown the post-MI remodeling is nearly completed in mice [8]. Colchicine had no effect on sham operated animals. However, colchicine injection prior to reperfusion significantly reduced QRS and QT duration (57.4 ± 4.4 vs 49.2 ± 8.1; n = 8; p = 0.01 and 63.7 ± 11.4 vs 50.2 ± 9.2; n = 8; p = 0.003, respectively). Furthermore, the HRV parameters such as SDNN (2.6 ± 0.75 vs 3.4 ± 0.69; 0.04; n = 8), TINN index (21.9 ± 8.8 vs 45.6 ± 24.6; p = 0.008; n = 8) as well as HRV index (1.1 ± 0.5 vs 1.5 ± 0.33; p = 0.05; n = 8) and RMSSD (3.7 ± 0.8 vs 5.2 ± 1.3; p = 0.05; n = 8) were improved compared with IR placebo group (Figure 1).

Here, the significant increase of QT interval observed after AMI is prevented under colchicine treatments. Colchicine may also regulate electrophysiological properties at the cellular level. Indeed, due to microtubule depolymerization properties and increase tubulin dimer, it was proposed that colchicine activates several G proteins which in turn may increase acetylcholine(Ach)-dependent K^+^ channels as well as L-type Ca^2+^ current conductance [9]. In addition, colchicine was also shown to prevent the impairment of t-tubule organization and excitation-contraction coupling in heart failure [10]. Altogether, these results may at least partly explain the reduced QT interval prolongation observed in the present study. Reduced HRV is a strong predictor of arrhythmic events compared with left ventricular ejection fraction reduction [11]. Colchicine injection restored all HRV parameters in our AMI model, indicating an improvement of the sympatho-vagal balance and therefore, a protective effect of colchicine on neuronal cells.

### Colchicine reduces sympathetic denervation in the remote area

**Figure 2.**
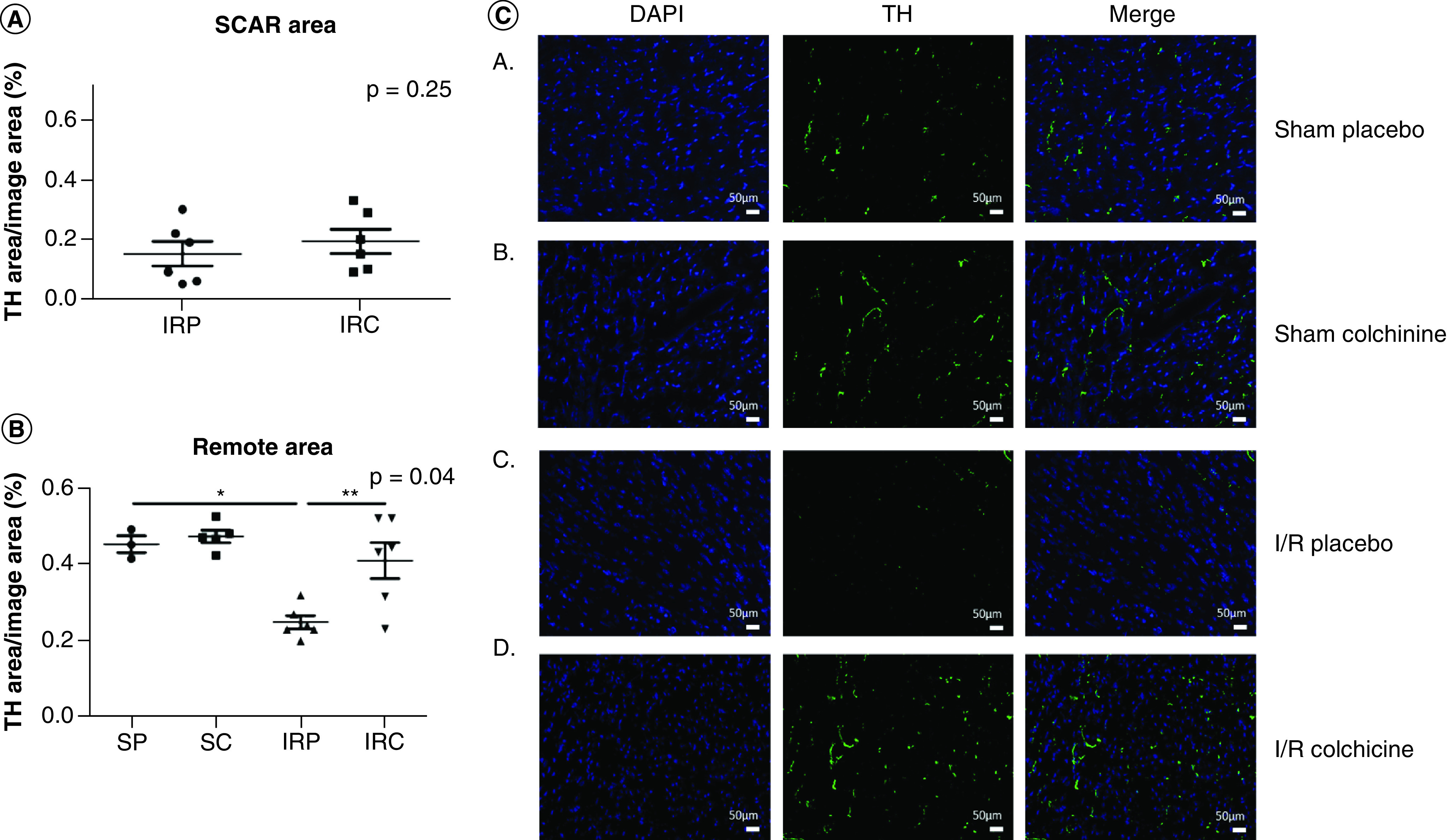
Sympathetic nerves quantification in the scar (A) and remote (B) areas of ischemic hearts on immune-fluorescence, with a typical example of remote area immunofluorescence (C). SP; SC; IRP; IRC; TH (specific antibody for sympathetic nerves immune-fixation); Note the important denervation in the remote area after myocardial infarction, reversed by Colchicine. *p < 0.05. IRC: IR colchicine; IRP: IR placebo; SC: Sham colchicine; SP: Sham placebo; TH: Tyrosine hydroxylase.

Then, in order to evaluate the myocardial sympathetic innervation, we performed tyrosine hydroxylase (TH) immunostaining in scar and remote areas 7 days after reperfusion (Figure 2). Colchicine treatment significantly preserved sympathetic innervation in the remote area in comparison with the animals treated by placebo (0.41 ± 0.11, n = 6 vs 0.24 ± 0.04, n = 6; p = 0.03), whereas in the scar area colchicine was ineffective (0.15 ± 0.10, n = 6 vs 0.19 ± 0.09, n = 6; p = 0.42).

These results demonstrate that colchicine prevents sympathetic denervation after AMI in the remote area. Sympathetic denervation of the nonischemic myocardium adjacent to the scar already proved to increase the risk of sudden death [12]. The perturbation of the neurotrophin balance is involved in the disruption of sympathetic nerves following AMI [1]. Mature NGF binds to the tropomyosin receptor kinase A (TrkA) receptor to mediate neurons survival and differentiation. On an opposite way, its precursor (protein pro-NGF), is elevated after AMI and promotes axon degeneration [13]. In addition, the brain-derived neurotrophic factor (BDNF) and Pro–BDNF also stimulate axon degeneration [14].

### Colchicine treatment prevents the increase in NGF serum concentration after AMI

We next evaluated the serum NGF concentration after AMI. Seven days after reperfusion NGF circulating level was significantly increased (p = 0.03; n = 5). This elevation was prevented by colchicine treatment prior reperfusion (p = 0.008; n = 5) (Supplementary Figure). No significant different was observed in sham-operated groups.

The increased NGF circulating level after AMI suggests a stimulation in neuronal growth to compensate for the denervation in the myocardium. Colchicine prevented this NGF blood level increase, likely due to a lower neuronal death.

## Conclusion

### Colchicine translation to bedsides applications

In addition to our previous study [5], new insights on colchicine molecular understanding are demonstrated here, paving the way for colchicine use in AMI. This is the golden age of anti-inflammatory therapies in myocardial infarction and atherosclerosis. The large Colchicine Cardiovascular Outcomes Trial (COLCOT) study demonstrated a significant reduction of ischemic cardiovascular events in the Colchicine group in comparison with the placebo [6]. Because of the low cost and the relative safety at low dose of colchicine, the COLCOT study [6] has a clinically-relevant application. Recently, the Low Dose Colchicine 2 (Lodoco2) trial demonstrated strong effect of colchicine in patients with stable coronary disease [15].

Our present results could provide an original explanation on cardiovascular mortality reduction with Colchicine. Based on this novel paradigm, the Colchicine to prevent myocardial Denervation after Myocardial Infarction (The COLD-MI trial – NCT04420264) will soon start and evaluate cardiac innervation 6 months after an AMI using myocardial ^123^I- méta-iodobenzylguanidine (MIBG) SPECT [16]. According to the present proof of concept study, COLD-MI may thus open important understanding in the cardioprotective effects of colchicine.

## Future perspective

Colchicine is arguably in the golden age of its utilization. Important results have been obtained in AMI, secondary prevention and stroke reduction. In the next few years, colchicine might even be tested in the primary prevention in high cardiovascular risk patients. We speculate colchicine could be included in the next STEMI, NSTEMI or prevention guidelines.

**Summary points**

- Acute myocardial infarction (AMI) was shown to induce local sympathetic denervation, predisposing to an electrical instability and arrhythmia in patients after AMI or in chronic heart failure.
- Colchicine could also prevent sympathetic denervation through anti-inflammatory pathway in a mice model of Ischemia/Reperfusion (I/R).
- I/R was induced in C57BL/6J male mice by temporary left coronary artery was ligated and 400 μg/kg of colchicine or the placebo was administrated before the reperfusion.
- We analyzed the NGF plasmatic level, ECG and heart rate variability (HRV) parameters and analyzed histologic sympathetic innervation in mice heart.
- Colchicine improves *in vivo* post ischemic ECG parameters and HRV at 7 days. Reduced HRV is a strong predictor of arrhythmic events. Colchicine injection restored all HRV parameters in our AMI model, indicating an improvement of the sympatho-vagal balance and therefore a protective effect of colchicine on neuronal cells.
- Colchicine reduces sympathetic denervation in the remote area. Sympathetic denervation of the nonischemic myocardium adjacent to the scar already proved to increase the risk of sudden death. No effect was observed in the scar area.
- Colchicine treatment prevents the increase in NGF serum concentration after AMI.
- We next evaluated the serum NGF concentration after AMI. Seven days after reperfusion NGF circulating level was significantly increased (p = 0.03; n = 5). This elevation was prevented by colchicine treatment prior reperfusion (p = 0.008; n = 5) (Supplementary Figure). No significant different was observed in sham operated groups.
- The increases NGF circulating level after AMI suggests a stimulation in neuronal growth to compensate for the denervation in the myocardium. Colchicine prevented this NGF blood level increase, likely due to a lower neuronal death.
- This is the golden age of anti-inflammatory therapies in myocardial infarction and atherosclerosis.
- Many trials (The large Colchicine Cardiovascular Outcomes Trial and the Low Dose Colchicine 2 (Lodoco2) trials demonstrated strong effect of colchicine in patients with AMI or stable coronary disease.
