# Supplementary figures and images for: Low-dose colchicine prevents sympathetic denervation after myocardial ischemia-reperfusion: a new potential protective mechanism
Source: Future Sci OA. 2020 Nov 23;7(2):FSO656. doi: 10.2144/fsoa-2020-0151 (PMC7787178; doi:10.2144/fsoa-2020-0151)

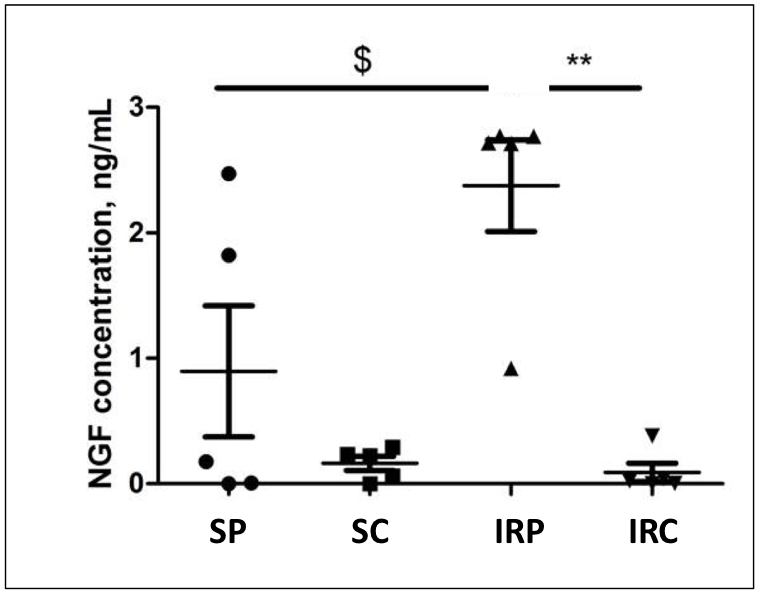

Supplement: Supplementary file 1 [file fsoa-07-656-s1.png]
